# Recognition of Basic Activities of Daily Living Using Wearable Devices for Older Adults: Scoping Review

**DOI:** 10.2196/67373

**Published:** 2025-05-15

**Authors:** Samantha J Ray, Josh Cherian, Amanda Mae Liberty, Tracy Anne Hammond, Paula K Shireman

**Affiliations:** 1 Department of Medical Physiology College of Medicine Texas A&M University Bryan, TX United States; 2 Center for Remote Health Monitoring, Department of Biomedical Engineering School of Medicine Wake Forest University Winston-Salem, NC United States; 3 Department of Computer Science & Engineering Texas A&M University College Station, TX United States; 4 Department of Primary Care & Rural Medicine College of Medicine Texas A&M University Bryan, TX United States

**Keywords:** activity recognition, health monitoring, wearable sensors, activities of daily living, aging in place

## Abstract

**Background:**

Tracking the performance of activities of daily living (ADLs) using ADL recognition has the potential to facilitate aging-in-place strategies, allowing older adults to live in their homes longer and enabling their families and caregivers to monitor changes in health status. However, the ADL recognition literature historically has evaluated systems in controlled settings with data from younger populations, creating the question of whether these systems will work in real-world conditions for older populations.

**Objective:**

This scoping review seeks to establish the state-of-the-art for recognizing basic ADLs using wearable sensors. This primary goal will identify literature gaps and research needed to make ADL tracking viable for aging-in-place solutions. In addition, this paper will quantify how many publications include older adults. This secondary goal assesses how often studies evaluate their system with older adult participants, enhancing the trustworthiness of the approach.

**Methods:**

We conducted a scoping review using the PRISMA-ScR (Preferred Reporting Items for Systematic reviews and Meta-Analyses extension for Scoping Reviews) guidelines. We identify studies focused on basic ADL recognition using wearable sensors within the PubMed, Association of Computing Machinery Digital Library (ACM DL), and Google Scholar databases using papers published in the last 5 calendar years (2019-2024) to identify current trends given the rapid changes in wearable technology devices. Publications must include at least one of the basic ADLs (ie, bathing, dressing, toileting, transferring, continence, and feeding) and include some sort of wearable sensor or device. Studies focusing on instrumental ADLs, general physical activity tracking, fall detection, or only using environmental devices are excluded. Studies that include older adults in the design or evaluation of their ADL recognition system are highlighted.

**Results:**

The database search identified 695 papers; 164 papers passed title screening. A total of 58 studies satisfied the inclusion criteria; only 8 studies included older adults despite most studies identifying this population as a focus for their research. Most studies focused on eating (n=27), hygiene (n=24), drinking (n=20), or transitions (n=13). Few works included toileting (n=3), dressing (n=2), or bathing (n=1) activities. Of the 8 studies that included older adults, 5 focused on recognition performance while 3 focused on user experience and system acceptability.

**Conclusions:**

Basic ADLs are unevenly covered in the literature; more research is needed for recognizing bathing, dressing, and toileting activities. Despite all studies stating the importance of tracking ADLs in older adults, only 14% (8/58) of the included works involve older adult participants. A commonality between these outcomes is difficulty collecting or obtaining adequate training data for ADL recognition systems. Many works are predominantly concerned with proving system feasibility and do not assess usability or real-world deployment. For these systems to move from academic experiments to actual systems with clinical utility, ADL recognition systems must consider the design requirements of being part of remote health monitoring systems.

## Introduction

The need for aging-in-place solutions increases as the population of adults over the age of 65 years escalates [1,2]. One way to enable people to live independently in their own homes is to use technology to aid health management. Existing examples of this type of technology include medication reminders, vital sign monitoring, and fall detection systems [3]. Many of these systems serve as safety nets to detect adverse events.

An open problem is developing systems that can automatically track the performance of activities of daily living (ADLs). ADLs are generally divided into 2 categories: basic and instrumental [4]. Basic ADLs (BADLs) are the essential activities to maintain quality of life and satisfy basic needs to stay alive [4-6]. By contrast, instrumental ADLs are characterized by more complex daily interactions, such as health and home management, driving and community mobility, child rearing, meal preparation and cleanup, medication management, and shopping [7]. The ability to perform ADLs determines whether a person can live independently. Tracking ADLs offers opportunities for remote health monitoring and proactive health care by detecting changes in ADL performance as early as possible. For example, ADL tracking can be used to predict the presence of an acute illness by detecting symptoms such as lethargy, weakness, and decreased appetite [8].

One way to automate ADL tracking is to use activity recognition, a subarea of artificial intelligence focused on understanding human behavior. Activity recognition has become more feasible given the commercial proliferation of sensors partnered with access to the sensor data as well as advances in machine learning techniques [9-11]. Early studies in the field relied on custom hardware for the recognition of human activities [12,13]. More recent work has predominantly used commercially available devices [9,10,14,15]. ADLs such as brushing teeth [16,17], taking medication [18,19], and washing hands [20] have been recognized with encouraging degrees of accuracy. Current research focuses on recognizing multiple ADLs to create ubiquitous health monitoring applications [10,21].

However, activity recognition covers a broad range of applications and hardware options. Novel sensors and approaches must collect custom datasets [9,10,22], but the standard practice is to evaluate proposed systems with existing datasets as benchmarks [11,21,23-29]. Publicly available datasets are heterogenous with respect to the activities included, sensors used, and placement of the sensors. However, a commonality is recruiting young, healthy adults, resulting in the average age generally being less than 30 years. Common benchmark datasets (eg, MobiAct [30], PAMAP2 [31], UniMiB-SHAR [32], and WISDM [33]) follow this trend with an average age of 27 years or less. Older adults are more likely to be included only if the application has strong ties to age (eg, fall detection with the SisFall dataset [34]).

Existing datasets often only include younger adults because the main impetus for most data collection is to prove system feasibility (ie, prove that a computer can recognize the targeted activities with the used sensors). Researchers often assume that systems trained on data from younger adults will perform adequately for older adults because researchers expect that their systems will generalize to new populations. However, the realization that this assumption does not hold is growing [35], and attention has been drawn specifically to activity recognition for older adults [36]. These systems need to be proven to be reliable when used by older adults prior to clinical use. Consequently, there exists an open question of how effective these ADL recognition systems would be as tools to support aging in place.

The primary goal of this scoping review is to understand the current state-of-the-art activity recognition systems focusing on BADLs using wearable devices. Recent, related reviews have focused on ambient sensors or smart home environments [37-40] or wearable sensors for just bathroom activities [41]. This primary goal will summarize trends in BADL recognition works and identify gaps in the literature. The secondary goal is to identify works that include older adults as participants. This secondary goal will give insight into how many of the identified works could be used in aging-in-place solutions. Our review seeks to answer the following questions:

1. What is the current state-of-the-art for recognizing BADLs using wearable sensors?
2. How many studies focused on BADL recognition using wearable sensors include older adults in the research?

## Methods

### Overview

This review identifies studies that focus on ADL recognition using wearable sensors to recognize basic ADLs as defined by Katz et al [5]. A subgoal within this review is to identify studies that include older adults in the research, for example, as participants in data collection for training ADL recognition systems or in user studies centered on using wearable sensors for ADL recognition. This work is a scoping review that follows the PRISMA-ScR (Preferred Reporting Items for Systematic Reviews and Meta-Analyses extension for Scoping Reviews) guidelines [42] and the protocol [43] is registered with the Open Science Foundation. Following standard procedures for PRISMA (Preferred Reporting Items for Systematic Reviews and Meta-Analyses), publications are systematically and hierarchically screened and assessed for eligibility. Title and abstracts are screened during the first step of the screening phase because some of our inclusion criteria (Table 1) may not appear in the title.

**Table 1:** Inclusion and exclusion criteria for paper selection.

Criteria type	Inclusion	Exclusion
Sensor type	Includes a sensor or device that is worn on the user’s body (ie, is a wearable solution)	Includes only sensors or devices that are placed in the environment (ie, is an ambient solution)
Included ADLs^a^	Includes at least one basic ADL (defined in Table 3)	Does not include any basic ADLs (defined in Table 3)
Paper focus	Focuses on ADL recognition or ADL tracking systems	Focuses on physical activity tracking (eg, exercise tracking) or fall detection
Publication date	Published in the range of January 1, 2019-December 31, 2024	Published before 2019

^a^ADL: activity of daily living.

### Sources of Evidence

The focus of this survey is relevant to both the medical and computer science literature. We queried both PubMed and the Association of Computing Machinery Digital Library (ACM DL) to survey a substantial corpus of studies in both domains. In addition, we supplemented the queries with additional studies identified from Google Scholar. The queries were conducted in January 2025.

### Search Strategy

The survey summarizes the state of basic ADL recognition by covering the last 5 calendar years (2019-2024). This timeframe focuses on recent trends as technology in this area can rapidly change, for example, the proliferation of consumer smart devices.

The search terms used in the queries are provided in Table 2. These terms align with previous reviews focused on ADL recognition [37,41]. Each row in Table 2 is combined with AND Boolean logic. “Wearable” is the only term used to specify the sensors used to minimize the capture of activity recognition systems using ambient or environmental sensors. The term “basic” is not clarified in the ADL-related terms because many works do not specify basic versus instrumental.

**Table 2:** Search terms used in database queries. Rows of search terms are combined with AND logic. The asterisk (*) is a special character to capture any number of additional characters.

Search terms	Rationale
Wearable	Use of wearable sensors or devices
elder* OR older	Target population of older adults
recogni* OR monitor* OR detect*	Focus on activity recognition
adl OR adls OR “activities of daily living”	Target within activity recognition

Katz et al [5] defines BADLs as Bathing, Dressing, Toileting, Transferring, Continence, and Feeding. The ability to perform these activities serves as a score of patient independence. The Katz Index of Independence in Activities of Daily Living gives clear definitions of what skills the patient needs to have and what types of assistance they can receive; for example, food preparation is not part of the Feeding activity. In practice, Katz BADLs are treated as categories and will include related and proxy activities. For example, Bathing includes other personal hygiene activities such as brushing teeth, and Toileting includes activities such as flushing which serve as a proxy indicator of the main activity. Continence is generally combined with Toileting as the former is not an activity within the scope of activity recognition research. Table 3 provides the adjusted definitions of each BADL to align with the practices in the ADL recognition literature.

Activity recognition has a distinct literature related to ambulation activities such as walking, running, and ascending or descending stairs. These studies are diverse with respect to focusing on clinical applications, for example, remote monitoring of rehabilitation after injury, or nonclinical applications, such as exercise tracking. Many of these studies model ambulation as different states of being (eg, “the person is currently sitting” or “the person is currently walking”) and do not capture transitions such as standing up from sitting. Katz’s definition of transferring focuses on transitions rather than the patient’s general mobility; we do not include general ambulation activities in Table 3. Recognition of ambulatory activities and related gait attributes merit their own study.

**Table 3:** Mapping of Katz's basic activities of daily living to activities.

BADL^a^ and categories		Example activities
Bathing		
	Bathing	Bathing and showering
	Hygiene	Brushing teeth and washing hands
Dressing		
	Dressing	Putting on and taking off clothing
Toileting and continence		
	Toileting	Using the toilet or urinal and flushing
Transferring		
	Transitions	Sitting-to-standing, lying-to-standing, and their inversions
Feeding		
	Eating	Eating with utensils and eating with hands
	Drinking	Drinking from a cup or mug

^a^BADL: basic activity of daily living.

The eligibility and exclusion criteria are given in Table 1. ADL recognition needs to be a direct outcome or goal of the study to be included. Furthermore, at least one BADL needs to be included; studies focused on instrumental ADLs such as cooking were excluded. Two related areas to BADL recognition are physical activity recognition and fall detection. Physical activity recognition papers tend to focus on maintaining one’s physical health and tracking exercise, which are disparate goals from basic ADL monitoring. Some studies in fall detection have the goal of distinguishing falls versus normal, daily activities, but the identification of daily activities is a strategy to decrease false positives in lieu of a direct goal of the work. As such, studies focused on general physical activity or fall detection are excluded.

The reviewing process was conducted predominantly by one author. The author met with at least 2 other authors to discuss ambiguous papers at the end of each step during the screening process. The eligibility and exclusion criteria were not subjective; one reviewer was sufficient for most papers.

## Results

### Overview

**Figure 1:**
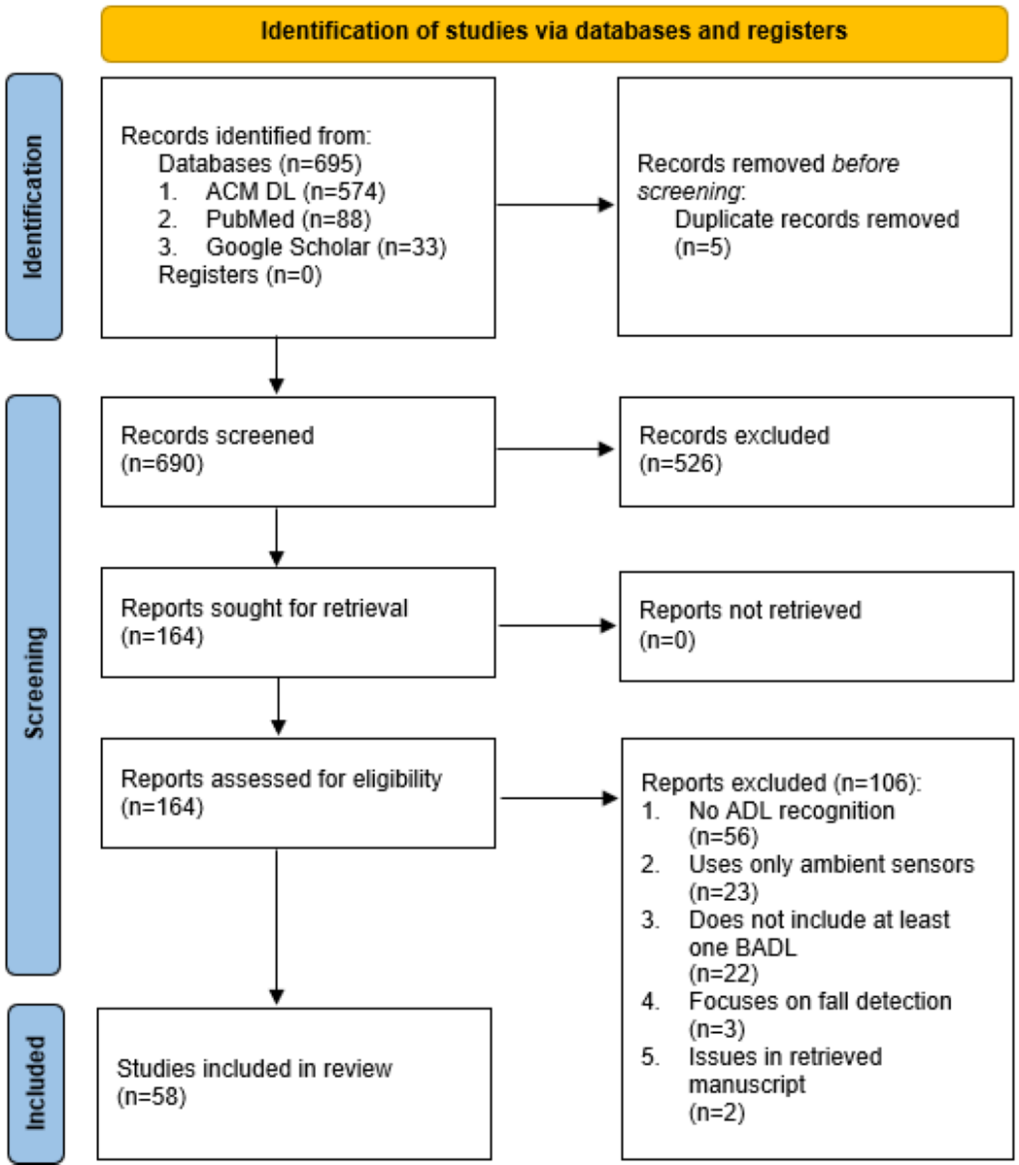
Paper selection flowchart based on PRISMA (Preferred Reporting Items for Systematic reviews and Meta-Analyses) guidelines. ACM DL: Association of Computing Machinery Digital Library; ADL: activity of daily living; BADL: basic activity of daily living.

The search resulted in 695 studies. After removing duplicates, the titles and abstracts of 690 publications were screened. A total of 164 studies were eligible for full-text assessment; 58 studies satisfied the inclusion criteria. Overall, 8 of these studies included older adults in the research. The paper selection flowchart is given in Figure 1.

We summarize the state-of-the-art for the recognition of BADLs in the following subsections (Table 4). We then highlight the papers that included older adults (Table 5).

Most studies use commodity devices such as smartphones and smartwatches for motion sensors and microphones regardless of which ADLs were targeted. Using these devices maximizes the solution’s potential for adoption as these devices are ubiquitous. Research using smartphones assumes that the phone will either be in the user's pocket or nearby them. Some studies strap the smartphone to a person’s arm to simulate a watch-like form factor [44] or to keep the device near the user in an unobtrusive manner [45]. Some studies still leverage custom sensor arrays, for example, the Opportunity dataset where participants had accelerometers placed all over the body [46] and the study by Bedri et al [47], which placed sensors on a pair of glasses, on the ear, and on the back of the neck.

**Table 4:** Activity of daily living recognition studies grouped by activities of daily living.

BADL^a^ and categories		Relevant studies	Number of studies
Bathing			
	Bathing	[45]	1
	Hygiene	[9,10,15,17,22,44,45,48-64]	24
Dressing			
	Dressing	[15,64]	2
Toileting and Continence			
	Toileting	[45,59,65]	3
Transferring			
	Transitions	[15,24-29,54,66-68]	13
Feeding			
	Eating	[10,15,22,24,47,55,57,58,60,62-64,69-83]	27
	Drinking	[9,10,15,22,24,55,58,59,62-64,67,69,74,77,83-87]	20

^a^BADL: basic activity of daily living.

### Bathing and Hygiene

Bathing and showering behaviors are rare inclusions for ADL recognition systems. Previous surveys did not capture any papers with these activities [37,41]. Liang et al [45] used an audio-based approach to detect sounds associated with bathtubs and showers, for example, filling with water or washing. The lack of focus on these activities has several likely reasons. First, these activities are difficult to simulate in laboratory settings due to their facility requirements. Second, wearable sensors need to be sufficiently waterproof or protected during these activities. Finally, people are more likely to be uncomfortable performing these activities while being recorded. The protocol in the study by Liang et al [45] avoided these issues. They conducted a free-living study where the researcher followed the participant around their own home, and the researcher kept a distance to minimize any influence on how activities were performed.

Many hygiene activities primarily involve the hands and can be captured with wrist-worn devices such as smartwatches. The most commonly included activities are brushing teeth, combing hair, and washing hands. Each of these activities tends to be performed for a sustained amount of time and has rhythmic attributes in their motions, making them distinct from other daily activities. Furthermore, these activities have low expected variation in their performance, making it easier for an ADL recognition model to generalize. These activities are usually included in a large set of general ADLs (ie, the work is not specifically focused on bathroom or hygiene activities). Examples of this practice include studies by Bhattacharya et al [10] and Cherian et al [22] that recognized 23 and 8 different activities, respectively, and covered a range of everyday activities. Exceptions to this pattern include Akther et al [17] who focused on assessing how thoroughly the user brushed their teeth and Mondol et al [51] and Santos-Gago et al [53] whose work focused on identifying hand-washing behaviors that are compliant with the World Health Organization (WHO) guidelines.

### Dressing

Dressing is an uncommon activity to be included within the constraints of this review. Motion sensors such as accelerometers are the most common type of sensor used in wearable systems. However, the motions associated with dressing activities tend to be subtle, making them difficult to distinguish from other everyday activities. Furthermore, the diversity in the styles of clothing people wear makes designing a general recognition system complicated. Sun et al [15] included “putting on clothes” and “taking off clothes” among the total of 221 activities included in their work focused on developing a multimodal activity recognition system. This work found that both motion and Wi-Fi signals were useful in determining if the user was interacting with clothing. Dressing in Narkhede et al [64] used both motion and location data and commented that location context was necessary due to the high variability in the motion data.

### Toileting

Toileting behaviors are generally detected via a proxy indicator. Using a toilet or urinal does not involve significant bodily motion, therefore common locations for wearable sensors will not detect these activities. However, this activity is normally followed by flushing the toilet or urinal, as this sound is used to signal the end of the activity. Liang et al [45] use only sound, Masum et al [65] uses only motion, and Mollyn et al [59] uses both. Liang et al [45] and Mollyn et al [59] specify that their systems recognize the flushing action while Masum et al [65] focuses on the action of sitting on the toilet.

### Transferring

Ambulation activities are widely studied in activity recognition research.

Many studies developed methods for distinguishing between different modes of ambulation and posture such as walking, jogging, ascending or descending stairs, sitting, standing, and lying down [88-93]. However, most of these studies focus on just detecting the current state of the user and do not directly capture the transitions between modes (eg, sit-to-stand, stand-to-sit, lying-to-stand, or stand-to-lying). The datasets UniMiB-SHAR [32], MobiAct [30], and Transitional Activities [94] do include these annotations, and many recent deep learning–centric studies have leveraged these datasets to detect these activities [11,24-29]. The first 2 datasets involve a smartphone placed in the front pocket of the user’s pants while the last dataset places sensor nodes across the body on the waist, right wrist, left wrist, right arm, left thigh, and right ankle.

### Eating and Drinking

Eating and drinking have the most diversity in the sensors used to recognize these activities. While they can be recognized with wrist-worn sensors [10,22,77], these activities also afford opportunities to place sensors on the head or neck [47,70,85]. Bedri et al [70] used a glasses form factor to detect episodes of eating and drinking. This approach allows the system to avoid ambiguity with other hand-centric activities and focus on detecting actions such as chewing and swallowing. Some studies will use the high-level labels in the Opportunity dataset [46] to recognize examples of eating and drinking. Opportunity contains kitchen-centric activities where the participant goes through a gauntlet of activities including opening and closing cabinets, opening and closing a refrigerator door, making a sandwich, and making coffee. This dataset has low-level annotations focused on the specific motions, for example, “open drawer” and high-level annotations focused on the activity being performed, such as “sandwich time.” Some studies only focus on recognizing the 17 gestures in an activity-agnostic fashion, but others use high-level annotations to recognize actions such as taking a bite of a sandwich or taking a sip of coffee [24,58,74,83].

### Studies With Older Adults

We highlight studies that include older adults in the main research (Table 5). Only 5 of these studies have the design and evaluation of activity recognition systems as their main contribution. The other 3 studies are highly connected to ADL recognition using wearable sensors, meriting their inclusion.

Alam et al [36] represents the concern that systems trained on younger populations will not generalize to older populations when deployed. Their work focuses on mitigating biases in their developed ADL recognition system to be robust to differences in physical ability. The main evaluation is a gesture recognition system to distinguish between 8 hand gestures and a walking recognition system that is robust to the user having a mobility aid such as a walker.

**Table 5:** Studies that include older adults.

Study type and references			Year		Focus
Activity recognition					
	Wellnitz et al [85]	2020		Drinking recognition	
	Alam et al [36]	2021		Bias mitigation	
	Cao et al [56]	2022		Handwashing recognition	
	Cook et al [57]	2022		Brain health intervention	
	Alevizaki et al [61]	2023		System design	
Human-computer interaction					
	Kim et al [95]	2022		In-situ data annotation system	
	Caldeira et al [96]	2023		User experience in smart home	
	Cherian et al [97]	2024		Acceptability of ADL monitoring system	

Cao et al [56] focuses on recognizing the activity of washing hands in older adults with dementia in a user-independent fashion. Their system identifies specific steps in the handwashing process to identify if the patient needs assistance in properly washing their hands. Their evaluation included 8 older adults with cognitive impairment as tested by the Montreal Cognitive Assessment (MoCA) [98].

Cook et al [57] leverages an ADL recognition system to label the participants’ behaviors in free-living conditions. The goal of this work was to distinguish between brain health intervention and nonintervention participants. The behavior predictions were used as input to the brain health intervention versus nonintervention classifier.

Kim et al [95] created a speech-based smartwatch application to gather in-situ annotations of daily activities. The design of the system focused on the needs and comfort of older adults to minimize the burden associated with data annotation. They conducted a user study over the course of 7 days with 13 older adults to evaluate the experience of using such a system. An envisioned goal for this system is facilitating personalized activity recognition.

Caldeira et al [96] and Cherian et al [97] include perspectives from older adults regarding the usage of monitoring technology for ADL performance. Calderia et al [96] interviewed participants after living in a smart home and wearing a smartwatch for 2.5 years. They found that participants wanted to be included and to engage with their data, especially with respect to determining if they were living an active lifestyle. Cherian et al [97] interviewed participants who lived in the assisted living section of a continuing care retirement community before and after wearing smartwatches for 1 week to simulate an ADL monitoring system. Participants acknowledged the potential utility of such a system and voiced a desire to maintain their independence.

Wellnitz et al [85] and Alevizaki et al [61] discuss that their systems have utility for ADL tracking for older adults and include at least one older adult in their data collection and system evaluation. However, the majority of their participants are younger adults, and including older adults was not an explicit goal.

## Discussion

### Principal Results

BADLs are not equally covered in the ADL recognition literature. Few studies attempt to recognize bathing, dressing, and toileting. These activities have attributes that make them more difficult to detect as stated in the Results section. Bathing and toileting relied on sound cues, and dressing relied on location context. By contrast, the other BADLs were recognizable using motion sensors, which are ubiquitous in commercially available wearable devices. The special facility requirements and concerns for subject privacy of bathing, toileting, and dressing may have caused them to be deprioritized in the ADL recognition literature because systems that recognize many activities (ie, multiclass recognition) are a current focus. However, recognizing bathing, dressing, and toileting has value in health monitoring applications as they give medical practitioners insight into their patients’ health, for example, whether the patient is maintaining regular bathroom habits. Robustly detecting these activities is an open problem for ADL recognition researchers to address as the field progresses.

All the papers collected in this scoping review discuss older adults at some point in their work by the nature of our query. However, our results show that only 14% (8/58) of studies in this scoping review include older adults in the design and evaluation of their systems. The main reason for this disconnect is that many publicly available datasets for ADL recognition include only younger adults [30-33,46]. Many techniques in artificial intelligence and machine learning require annotated datasets, and researchers are strongly encouraged to use existing datasets to benchmark their contributions and increase the reproducibility of their work. Including older adults requires conducting a custom user study and annotating the data which is time-consuming and costly. Evaluating the system with the target audience is not a necessary condition for the completion of studies that focus on developing new systems or techniques, for example, exploring self-supervised learning. This type of research consequently remains relatively unexplored, creating a knowledge gap in how well systems trained on younger populations generalize to older populations.

### Comparison to Prior Work

Activity recognition research is united by common goals. Techniques and approaches vary greatly across works, making surveys valuable summaries of the myriad of explored solutions. The most related scoping reviews to this work are the Camp et al survey of tools for ADL tracking for community-dwelling older adults [37] and Zhang et al survey of recognition of bathroom activities with wearable devices [41]. Camp et al did not focus on the sensing medium (ie, wearable versus ambient) but instead centered on commercially available devices. The study scope of Zhang et al was limited to only bathroom activities which are understudied as supported by this work. The current contribution is distinct by focusing on summarizing the state-of-the-art research approaches for all BADLs.

### Limitations and Future Directions

One of the goals of this work was to summarize the current practices in the design of ADL recognition systems for BADLs. A limitation of the search strategy used in this review is the inability to establish a performance benchmark for future research. We summarize the common techniques and considerations the studies contribute, but the performance evaluations of their solutions were not captured due to the heterogeneity of the data.

Another limitation is that our approach does not provide concrete insights into how to incorporate wearable ADL tracking systems into aging-in-place solutions. The studies in this survey predominantly focus on proving system feasibility, not usability. Understanding what older adults, their caregivers, and their loved ones desire in an ADL tracking system for supporting aging in place is an avenue for future research.

Our work focused on ADL tracking systems for BADLs to support aging in place. Future surveys should consider systems for recognizing instrumental ADLs or other solutions for supporting aging in place. Potential avenues include intelligent systems for home automation and social robotics for assistance in performing ADLs.

Most systems in activity recognition are evaluated in terms of correctly identifying the activity performed. However, this problem definition ignores the additional information about how the activity was performed (ie, whether it was performed correctly, adequately, or even abnormally). Systems that can detect and assess activity performance have more utility for caregivers and family members, providing more insight into the individual's health status. Specifically, knowing the quality of the activity performance can be used to detect declines in health and signal the need for an intervention or increase in needed care. Designing ADL recognition systems that can detect degradation in ADL performance is a potential avenue for future work.

### Conclusions

Over the coming decades, the population of older adults is expected to increase significantly, a trend that will put tremendous strain on health care systems around the world. Making it possible for people to stay in their homes longer safely (ie, aging in place) has great potential clinical implications. One solution to support aging in place is using human activity recognition systems to automatically track ADL performance, providing a safety net that can detect significant changes in ADL performance.

Wearable ADL recognition has promise for enabling these aging-in-place systems, but the current literature has several gaps to be addressed before this option becomes feasible. Several basic ADLs (eg, bathing, dressing, and toileting) have little coverage and remain open problems for ADL recognition. Additionally, many works are predominantly concerned with proving system feasibility and do not assess usability or real-world deployment. For these systems to move from academic experiments to actual systems with clinical utility, ADL recognition systems must consider the design requirements of being part of remote health monitoring systems.

In this survey, we reviewed human activity recognition systems designed to recognize basic ADLs using wearable sensors. Despite targeting older adults as users, many studies do not directly include this population in their research. To address this gap, ADL recognition researchers are encouraged to evaluate their systems with older adults as participants to assess how their systems would work in a real-world deployment.

## Appendix

Multimedia Appendix 1 PRISMA-ScR Checklist.

## Abbreviations

ACM DL: Association of Computing Machinery Digital Library
ADL: activity of daily living
BADL: basic activity of daily living
MoCA: Montreal Cognitive Assessment
PRISMA: Preferred Reporting Items for Systematic reviews and Meta-Analyses
PRISMA-ScR: Preferred Reporting Items for Systematic reviews and Meta-Analyses extension for Scoping Reviews
WHO: World Health Organization

## References

[1] (2017). World Population Prospects: The 2017 Revision.

[2] (2015). World Population Ageing.

[3] Haufe M, Peek STM, Luijkx KG (2019). Matching gerontechnologies to independent-living seniors' individual needs: development of the GTM tool. BMC Health Serv Res.

[4] Costenoble A, Knoop V, Vermeiren S, Vella R A, Debain A, Rossi G, Bautmans I, Verté D, Gorus E, De Vriendt P (2021). A comprehensive overview of activities of daily living in existing frailty instruments: a systematic literature search. Gerontologist.

[5] Katz S (1983). Assessing self-maintenance: activities of daily living, mobility, and instrumental activities of daily living. J Am Geriatr Soc.

[6] Edemekong PF, Bomgaars DL, Sukumaran S, Schoo C (2023). Activities of Daily Living.

[7] Amini DA, Kannenberg K, Bodison S, Chang PFJ (2014). Occupational therapy practice framework: domain and process (3rd edition). Am J Occup Ther.

[8] Boockvar KS, Lachs MS (2003). Predictive value of nonspecific symptoms for acute illness in nursing home residents. J Am Geriatr Soc.

[9] Laput G, Harrison C (2019). Sensing fine-grained hand activity with smartwatches.

[10] Bhattacharya S, Adaimi R, Thomaz E (2022). Leveraging sound and wrist motion to detect activities of daily living with commodity smartwatches.

[11] Haresamudram H, Essa I, Plötz T (2022). Assessing the state of self-supervised human activity recognition using wearables.

[12] Bao L, Intille SS (2004). Activity recognition from user-annotated acceleration data.

[13] Kao TP, Lin CW, Wang JS (2009). Development of a portable activity detector for daily activity recognition.

[14] Shoaib M, Bosch S, Incel O, Scholten H, Havinga P (2016). Complex human activity recognition using smartphone and wrist-worn motion sensors. Sensors (Basel).

[15] Sun K, Xia C, Zhang X, Chen H, Zhang CJ (2024). Multimodal daily-life logging in free-living environment using non-visual egocentric sensors on a smartphone.

[16] Cherian J, Rajanna V, Goldberg D, Hammond T (2017). Did you remember to brush?: a noninvasive wearable approach to recognizing brushing teeth for elderly care.

[17] Akther S, Saleheen N, Saha M, Shetty V, Kumar S (2021). mTeeth: identifying brushing teeth surfaces using wrist-worn inertial sensors.

[18] Kalantarian H, Alshurafa N, Sarrafzadeh M (2016). Detection of gestures associated with medication adherence using smartwatch-based inertial sensors. IEEE Sens J.

[19] Cherian J, Ray S, Hammond T (2021). An activity recognition system for taking medicine using in-the-wild data to promote medication adherence.

[20] Galluzzi V, Herman T, Polgreen P (2015). Hand hygiene duration and technique recognition using wrist-worn sensors.

[21] Das D, Nishimura Y, Vivek RP, Takeda N, Fish ST, Plötz T, Chernova S (2023). Explainable activity recognition for smart home systems. ACM Trans. Interact. Intell. Syst.

[22] Cherian J, Ray S, Taele P, Koh JI, Hammond T (2024). Exploring the impact of the NULL class on in-the-wild human activity recognition. Sensors (Basel).

[23] Abedin A, Ehsanpour M, Shi Q, Rezatofighi H, Ranasinghe DC (2021). Attend and discriminate: Beyond the state-of-the-art for human activity recognition using wearable sensors.

[24] Rokni SA, Ghasemzadeh H (2019). Share-n-learn: a framework for sharing activity recognition models in wearable systems with context-varying sensors. ACM Trans. Des. Autom. Electron. Syst.

[25] Saeed A, Ozcelebi T, Lukkien J (2019). Multi-task self-supervised learning for human activity detection. Proc. ACM Interact. Mob. Wearable Ubiquitous Technol.

[26] Haresamudram H, Essa I, Plötz T (2021). Contrastive predictive coding for human activity recognition. Proc. ACM Interact. Mob. Wearable Ubiquitous Technol.

[27] Lu W, Wang J, Chen Y, Pan SJ, Hu C, Qin X (2022). Semantic-discriminative mixup for generalizable sensor-based cross-domain activity recognition. Proc. ACM Interact. Mob. Wearable Ubiquitous Technol.

[28] Zhang Y, Wang L, Chen H, Tian A, Zhou S, Guo Y (2022). IF-ConvTransformer: a framework for human activity recognition using IMU fusion and ConvTransformer. Proc. ACM Interact. Mob. Wearable Ubiquitous Technol.

[29] Kang H, Hu Q, Zhang Q (2024). SF-Adapter: Computational-efficient source-free domain adaptation for human activity recognition. Proc. ACM Interact. Mob. Wearable Ubiquitous Technol.

[30] Chatzaki C, Pediaditis M, Vavoulas G, Tsiknakis M, Röcker C, O'Donoghue J, Ziefle M, Helfert M, Molloy W (2017). Human daily activity and fall recognition using a smartphone’s acceleration sensor. Information and Communication Technologies for Ageing Well and E-Health.

[31] Reiss A, Stricker D (2012). Introducing a new benchmarked dataset for activity monitoring.

[32] Micucci D, Mobilio M, Napoletano P (2017). UniMiB SHAR: A dataset for human activity recognition using acceleration data from smartphones. Appl Sci.

[33] Weiss GM, Yoneda K, Hayajneh T (2019). Smartphone and smartwatch-based biometrics using activities of daily living. IEEE Access.

[34] Sucerquia A, López JD, Vargas-Bonilla JF (2017). SisFall: a fall and movement dataset. Sensors (Basel).

[35] Mehrabi N, Morstatter F, Saxena N, Lerman K, Galstyan A (2021). A survey on bias and fairness in machine learning. ACM Comput. Surv.

[36] Alam MAU (2021). AI-Fairness towards activity recognition of older adults.

[37] Camp N, Lewis M, Hunter K, Johnston J, Zecca M, Di Nuovo A, Magistro D (2020). Technology used to recognize activities of daily living in community-dwelling older adults. Int J Environ Res Public Health.

[38] Morita PP, Sahu KS, Oetomo A (2023). Health monitoring using smart home technologies: scoping review. JMIR Mhealth Uhealth.

[39] Facchinetti G, Petrucci G, Albanesi B, De Marinis MG, Piredda M (2023). Can smart home technologies help older adults manage their chronic condition? A systematic literature review. Int J Environ Res Public Health.

[40] Tannou T, Lihoreau T, Couture M, Giroux S, Wang RH, Spalla G, Zarshenas S, Gagnon-Roy M, Aboujaoudé A, Yaddaden A, Morin L, Bier N (2023). Is research on 'smart living environments' based on unobtrusive technologies for older adults going in circles? Evidence from an umbrella review. Ageing Res Rev.

[41] Zhang Y, D'Haeseleer I, Coelho J, Vanden Abeele V, Vanrumste B (2021). Recognition of bathroom activities in older adults using wearable sensors: a systematic review and recommendations. Sensors (Basel).

[42] Tricco AC, Lillie E, Zarin W, O'Brien KK, Colquhoun H, Levac D, Moher D, Peters MD, Horsley T, Weeks L, Hempel S, Akl EA, Chang C, McGowan J, Stewart L, Hartling L, Aldcroft A, Wilson MG, Garritty C, Lewin S, Godfrey CM, Macdonald MT, Langlois EV, Soares-Weiser K, Moriarty J, Clifford T, Tunçalp Ö, Straus SE (2018). PRISMA extension for scoping reviews (PRISMA-ScR): checklist and explanation. Ann Intern Med.

[43] Ray S (2024). Recognition of basic ADLs using wearable sensors. OSF.

[44] Abreu M, Barandas M, Leonardo R, Gamboa H (2019). Detailed human activity recognition based on multiple HMM.

[45] Liang D, Thomaz E (2019). Audio-Based Activities of Daily Living (ADL) recognition with large-scale acoustic embeddings from online videos. Proc. ACM Interact. Mob. Wearable Ubiquitous Technol.

[46] Chavarriaga R, Sagha H, Calatroni A, Digumarti ST, Tröster G, Millán JDR, Roggen D (2013). The opportunity challenge: a benchmark database for on-body sensor-based activity recognition. Pattern Recognition Letters.

[47] Bedri A, Liang Y, Boovaraghavan S, Kaufman G, Goel M (2022). FitNibble: A field study to evaluate the utility and usability of automatic diet monitoring in food journaling using an eyeglasses-based wearable.

[48] Akther S, Saleheen N, Samiei SA, Shetty V, Ertin E, Kumar S (2019). mORAL: An mHealth model for inferring oral hygiene behaviors in-the-wild using wrist-worn inertial sensors. Proc. ACM Interact. Mob. Wearable Ubiquitous Technol.

[49] Luo C, Feng X, Chen J, Li J, Xu W, Li W (2019). Brush like a dentist: Accurate monitoring of toothbrushing via wrist-worn gesture sensing.

[50] Hussain Z, Waterworth D, Aldeer M, Zhang W, Sheng Q (2020). Toothbrushing data and analysis of its potential use in human activity recognition applications: dataset.

[51] Mondol MAS, Stankovic JA (2020). HAWAD: Hand washing detection using wrist wearable inertial sensors.

[52] Samyoun S, Shubha SS, Sayeed Mondol MA, Stankovic JA (2021). iWash: A smartwatch handwashing quality assessment and reminder system with real-time feedback in the context of infectious disease. Smart Health (Amst).

[53] Santos-Gago JM, Ramos-Merino M, Alvarez-Sabucedo LM (2021). Identification of free and WHO-compliant handwashing moments using low cost wrist-worn wearables. IEEE Access.

[54] Xia C (2021). Optimal sensor position: Exploring the interface between the user and sensor in activity recognition system.

[55] Bhalla S, Goel M, Khurana R (2021). IMU2Doppler: Cross-Modal domain adaptation for doppler-based activity recognition using IMU data. Proc. ACM Interact. Mob. Wearable Ubiquitous Technol.

[56] Cao Y, Li F, Chen H, Liu X, Yang S, Wang Y (2022). Leveraging wearables for assisting the elderly with dementia in handwashing. IEEE Trans. on Mobile Comput.

[57] Cook DJ, Strickland M, Schmitter-Edgecombe M (2022). Detecting smartwatch-based behavior change in response to a multi-domain brain health intervention. ACM Trans Comput Healthc.

[58] Liang D, Li G, Adaimi R, Marculescu R, Thomaz E (2022). AudioIMU: Enhancing inertial sensing-based activity recognition with acoustic models.

[59] Mollyn V, Ahuja K, Verma D, Harrison C, Goel M (2022). SAMoSA: Sensing activities with motion and subsampled audio. Proc. ACM Interact. Mob. Wearable Ubiquitous Technol.

[60] Woodward K, Kanjo E, Taylor K, Hunt J (2022). A multi-sensor deep learning approach for complex daily living activity recognition.

[61] Alevizaki A, Pham N, Trigoni N (2023). Invited paper: Hierarchical activity recognition with smartwatch IMU.

[62] Schleter B, Avdonina M, Adhikary R, Jaisinghani D, Sen S (2024). Poster: An automated method to detect tooth brushing activity with smartwatch sensors.

[63] Mahmud S, Parikh V, Liang Q, Li K, Zhang R, Ajit A, Gunda V, Agarwal D, Guimbretiere F, Zhang C (2024). ActSonic: recognizing everyday activities from inaudible acoustic wave around the body. Proc. ACM Interact. Mob. Wearable Ubiquitous Technol.

[64] Narkhede A, Gowing H, Vandenberg T, Phan S, Wong J, Chan A (2024). Automated detection of in-home activities with ultra-wideband sensors. Sensors (Basel).

[65] Masum AKM, Jannat S, Bahadur EH, Alam MGR, Khan SI, Alam MR (2019). Human activity recognition using smartphone sensors: a dense neural network approach.

[66] Tang CI, Perez-Pozuelo I, Spathis D, Brage S, Wareham N, Mascolo C (2021). SelfHAR: Improving human activity recognition through self-training with unlabeled data.

[67] Augustinov G, Nisar M, Li F, Tabatabaei A, Grzegorzek M, Sohrabi K, Fudickar S (2023). Transformer-based recognition of activities of daily living from wearable sensor data.

[68] Wang S, Wang J, Xi H, Zhang B, Zhang L, Wei H (2024). Optimization-free test-time adaptation for cross-person activity recognition.

[69] Gomes D, Mendes-Moreira J, Sousa I, Silva J (2019). Eating and drinking recognition in free-living conditions for triggering smart reminders. Sensors (Basel).

[70] Bedri A, Li D, Khurana R, Bhuwalka K, Goel M (2020). FitByte: Automatic diet monitoring in unconstrained situations using multimodal sensing on eyeglasses.

[71] Zhang S, Zhao Y, Nguyen DT, Xu R, Sen S, Hester J, Alshurafa N (2020). NeckSense: a multi-sensor necklace for detecting eating activities in free-living conditions.

[72] Akbari A, Grimsley R, Jafari R (2021). Data-driven context detection leveraging passively sensed nearables for recognizing complex activities of daily living. ACM Trans. Comput. Healthcare.

[73] Kyritsis K, Diou C, Delopoulos A (2021). A data driven end-to-end approach for in-the-wild monitoring of eating behavior using smartwatches. IEEE J. Biomed. Health Inform.

[74] Lago P, Matsuki M, Adachi K, Inoue S (2021). Using additional training sensors to improve single-sensor complex activity recognition.

[75] Morshed MB, Bin MM, Haresamudram HK, Bandaru D, Abowd GD, Ploetz T (2022). A personalized approach for developing a snacking detection system using earbuds in a semi-naturalistic setting.

[76] Saphala A, Zhang R, Amft O (2022). Proximity-based eating event detection in smart eyeglasses with expert and data models.

[77] Staab S, Bröning L, Luderschmidt J, Martin L (2022). Performance comparison of motion-related sensor technology and acoustic sensor technology in the field of human health monitoring.

[78] Wang L, Allman-Farinelli M, Yang JA, Taylor JC, Gemming L, Hekler E, Rangan A (2022). Enhancing nutrition care through real-time, sensor-based capture of eating occasions: a scoping review. Front Nutr.

[79] Zhang R, Zhang J, Gade N, Cao P, Kim S, Yan J, Zhang C (2022). EatingTrak: Detecting fine-grained eating moments in the wild using a wrist-mounted IMU.

[80] Assi K, Meegahapola L, Droz W, Kun P, Götzen AD, Bidoglia M, Stares S, Gaskell G, Chagnaa A, Ganbold A (2023). Complex daily activities, country-level diversity,smartphone sensing: a study in denmark, italy, mongolia, paraguay, and UK.

[81] Hiraguchi H, Perone P, Toet A, Camps G, Brouwer A (2023). Technology to automatically record eating behavior in real life: a systematic review. Sensors (Basel).

[82] Pedram M, Fernandes G, Romano C, Wei B, Sen S, Hester J, Alshurafa N (2023). Experience: barriers and opportunities of wearables for eating research.

[83] Kianpisheh M, Mariakakis A, Truong KN (2024). exHAR: An interface for helping non-experts develop and debug knowledge-based human activity recognition systems.

[84] Gómez-Carmona O, Casado-Mansilla D, López-de-Ipiña D, García-Zubia J (2019). Simplicity is best: Addressing the computational cost of machine learning classifiers in constrained edge devices.

[85] Wellnitz A, Wolff JP, Haubelt C, Kirste T (2020). Fluid intake recognition using inertial sensors.

[86] Wang C, Kumar TS, De RW, Camps G, Hallez H, Vanrumste B (2022). Drinking gesture detection using wrist-worn IMU sensors with multi-stage temporal convolutional network in free-living environments.

[87] Hsieh CY, Huang HY, Chan CT, Chiu LT (2023). An analysis of fluid intake assessment approaches for fluid intake monitoring system. Biosensors (Basel).

[88] Pärkkä J, Ermes M, Korpipää P, Mäntyjärvi J, Peltola J, Korhonen I (2006). Activity classification using realistic data from wearable sensors. IEEE Trans Inf Technol Biomed.

[89] Zhu C, Sheng W (2009). Human daily activity recognition in robot-assisted living using multi-sensor fusion.

[90] Ronao CA, Cho SB (2016). Human activity recognition with smartphone sensors using deep learning neural networks. Expert Systems with Applications.

[91] Weiss GM, Timko JL, Gallagher CM, Yoneda K, Schreiber AJ (2016). Smartwatch-based activity recognition: a machine learning approach.

[92] Qin Z, Zhang Y, Meng S, Qin Z, Choo KKR (2020). Imaging and fusing time series for wearable sensor-based human activity recognition. Information Fusion.

[93] Huang W, Zhang L, Wang S, Wu H, Song A (2022). Deep ensemble learning for human activity recognition using wearable sensors via filter activation. ACM Trans. Embed. Comput. Syst.

[94] Ghasemzadeh H, Amini N, Saeedi R, Sarrafzadeh M (2015). Power-aware computing in wearable sensor networks: an optimal feature selection. IEEE Trans. on Mobile Comput.

[95] Kim YH, Chou D, Lee B, Danilovich M, Lazar A, Conroy DE, Kacorri H, Choe EK (2022). MyMove: facilitating older adults to collect in-situ activity labels on a smartwatch with speech.

[96] Caldeira C, Nurain N, Heintzman AA, Molchan H, Caine K, Demiris G, Siek KA, Reeder B, Connelly K (2023). How do i compare to the other people?": Older Adults' perspectives on personal smart home data for self-management".

[97] Cherian J, Ray S, Mernar T, Taele P, Mach H, Koh JI, Ye P, Hammond T (2024). A step toward better care: understanding what caregivers and residents in assisted living facilities value in health monitoring systems.

[98] Nasreddine ZS, Phillips NA, Bédirian V, Charbonneau S, Whitehead V, Collin I, Cummings JL, Chertkow H (2005). The montreal cognitive assessment, MoCA: a brief screening tool for mild cognitive impairment. J Am Geriatr Soc.

